# Engaging Older Adolescent Boys Into School-Based Mental Health Workshops: Testing Theory-Based Facilitators and Barriers in Focus Groups

**DOI:** 10.1177/15579883231177975

**Published:** 2023-10-11

**Authors:** Stephen Lisk, Ilyas Sagar-Ouriaghli, Ben Carter, Irene Sclare, Jennifer Holly, June S. L. Brown

**Affiliations:** 1 Department of Psychology, Institute of Psychiatry, Psychology & Neuroscience, King’s College London, London, UK; 2 Department of Biostatistics and Health Informatics, Institute of Psychiatry Psychology and Neuroscience, King’s College London, London, UK; 3 Southwark CAMHS Clinical Academic Group, South London & Maudsley NHS Foundation Trust, London, UK

**Keywords:** facilitators, barriers, help-seeking, adolescents, mental health

## Abstract

Untreated mental health problems continue from childhood and adolescence into adulthood, meaning accessible early intervention is essential to reduce long-term negative outcomes. However, there is often a reluctance to engage in mental health treatment, with considerable evidence that young men are less likely to seek help than young women. This original research study aimed to explore four areas of interest around facilitating engagement of adolescent boys to a stress workshop intervention for adolescents in U.K. schools. The areas explored were male role models, destigmatizing language, trust building, and using a transparent and collaborative approach. We also sought to understand the main barriers to engagement. To explore these areas of interest, two focus groups were run, with a total of 12 young men, over two regional sites (London and Bath). Content analysis was used to analyze the data. Participants particularly valued transparency and collaboration as strong facilitators to engagement. Building of trust was the next most popular. Use of role models and destigmatizing language were the joint third most popular methods. The main barrier to help-seeking identified was perceived threat to masculine identity (self and social stigma). Given these novel findings, the factors of transparency and collaboration and building trust as facilitators merit further research, among both adults and adolescents.

## Introduction

For a significant portion of men, mental health problems in childhood and adolescence continue into adulthood if left untreated (Odgers et al., 2007). However, there is consistent evidence that men are reluctant to seek help from health professionals (Sierra Hernandez et al., 2014; Slade et al., 2009). In particular, seeking help for mental health problems is significantly less common in men than women (Andrews et al., 2001; McManus et al., 2016; Yousaf et al., 2015). A 2020 government review in the United Kingdom found that one in six individuals aged 16 and above reported experiencing symptoms of a common mental disorder (CMD) within the past week. CMDs were more commonly reported by women than men across all age categories, with the highest prevalence among those aged 16 to 24. Women were also more likely than men to enter treatment after referral and to finish a course of treatment (Baker, 2020). Of note, there is also evidence suggesting boys start to disengage from health services around the time of adolescence (Marcell et al., 2002; Mason-Jones et al., 2012), which is also a significant period of onset for some mental health disorders (Beesdo et al., 2009). Therefore, there is a clear need to improve upon our current success in encouraging young men to seek and sustain help for their mental health.

Although there is a significant body of literature on help-seeking behavior in adult men with mental health problems, there is limited research on this topic in adolescents (Rice et al., 2018; Sagar-Ouriaghli et al., 2019; Seidler et al., 2016, 2019). However, it is crucial to understand the patterns of help-seeking behavior in adolescents, considering 50% of mental health problems occur by age 14, and 75% by age 24 (Kessler et al., 2007). In light of this, it is pertinent to begin by presenting the existing literature on key theoretical accounts of barriers and facilitators to help-seeking in adult and younger men, as this knowledge will serve as a foundation for formulating effective strategies to engage adolescent young men in seeking help for their mental health concerns.

*Conformity to masculine norms* has been heavily implicated in reducing help-seeking behaviors of men. Particularly within Western cultures, boys are socialized to embody “traditional” masculine roles that actively discourage vulnerability and weakness (Evans et al., 2011; Tang et al., 2014). In a recent review of the literature, Seidler and colleagues (2016) reported that conformity to masculine norms had a negative effect on men’s expression and management of symptoms, as well as their attitudes to, intention toward, and actual help-seeking behavior. *Self and social stigma* have also been identified as barriers to help-seeking: Feelings of *shame* can be seen as intertwined with self-stigma and the threat posed to one’s masculine identity when confronted with the prospect of seeking help for mental ill-health. Social stigma has identified as having greater prevalence in men compared with women (Corrigan & Watson, 2007; Latalova et al., 2014). Thus, self-stigma, and associated feelings of shame, can often result in avoidance of help-seeking and (consequently) negative long-term health outcomes (Rice et al., 2016; Sorsoli et al., 2008). Research with adolescent boys has identified shame (and a need to protect their reputation) as a significant barrier to seeking help-seeking (Rice et al., 2016). Research indicates that adolescent boys and young men may experience feelings of shame more intensely than girls or young women when their masculine identity is threatened (Vandello et al., 2008). Vandello and Bosson (2013) propose that manhood is viewed as a precarious social status that is challenging to attain and maintain, leading to maladaptive behaviors to uphold their gender status and avoidance of beneficial behaviors like help-seeking.

Young men struggle with *mental health symptom recognition and literacy* compared with young women (Rice et al., 2018). Cotton et al (2006) reported that when interviewing 1,207 young people between 12 and 25 years of age, female respondents (60.7%) were significantly more likely to correctly identify depression in a vignette compared with male respondents (34.5%). In addition, mental health literacy can also be considered as the understanding and awareness of mental health services (Jorm, 2000). There is significant evidence to suggest that the ability to identify mental health symptoms does not always translate to help-seeking behavior (Bonabi et al., 2016; Thompson et al., 2004). The ability to connect one’s experience to an appropriate service is equally as important (Bonabi et al., 2016; Thompson et al., 2004). Research has reported this knowledge of relevant services to be lower in both men (Lynch et al., 2018; Rafal et al., 2018) and adolescents (Attygalle et al., 2017).

Several approaches have now been proposed within the research literature to address these challenges. These can be identified as:

### Normalizing Help-Seeking With Positive Masculinity and Utilization of Role Models

Conforming to masculine norms may hinder help-seeking, but some “masculine” traits can be useful in mental health treatment. Kiselica and Englar-Carlson (2010) propose a positive psychology/positive masculinity framework that emphasizes male strengths and traditional male approaches like problem-solving, rather than “deficit models” of masculinity (which emphasize what is “wrong” with men). This view is also supported by prior research: O’Brien et al. (2005) reported that help-seeking was more readily embraced when it aligned with healthy masculine ideals. Emphasizing positive outcomes and aligning with healthy masculine ideals could improve engagement in men’s mental health treatment.

Using positive male *role models* or *aspirational figures* can help normalize and destigmatize help-seeking. A review by Sagar-Ouriaghli et al. (2019) highlighted the use of role models as a behavior change technique within male-specific interventions for promoting psychological help-seeking. Role models can normalize problems, model positive outcomes, and offer evidence that difficulties experienced are often simply the result of everyday stressors. Celebrities used as narratives in teaching about mental illness can also reduce stigma and increase help-seeking (Ferrari, 2016). Parents also play a crucial role, with young people’s attitudes toward mental health often influenced by those of their parents (Jorm & Wright, 2008). Adolescents are also more likely to seek help for mental health when they have an engaged and supportive father figure (Reeb & Conger, 2011).

### Tailoring Language to Destigmatize and Normalize Help-Seeking

Language has been demonstrated as central when relating with and engaging male clients in mental health treatment, with suggestions that more purposeful *self-disclosure* and use of colloquial and *de-stigmatizing language* may help improve male engagement and retention (Brown et al., 2004; Mahalik et al., 2012). Furthermore, utilizing colloquial and de-stigmatizing language may improve recognition of symptoms among boys who have previously failed to understand the applicability of these types of interventions to their own emotional experiences. Indeed, this may help to engage young boys who have lower mental health literacy as colloquial terms are more readily understood.

### Trust Building

While apprehension and caution are common in young men when discussing mental health (O’Brien et al., 2005; Vogel et al., 2011), building trust is crucial for engaging young men in mental health discussions and can be achieved through strategies such as emphasizing confidentiality (Kapphahn et al., 1999) or adopting a collaborative approach (Seidler, Rice, Oliffe, et al., 2018). Another novel approach to trust building, particularly in a school-based group therapy format, is the use of non-specific spaces inspired by the “men’s sheds” model (Milligan et al., 2013), as investigated by Sagar-Ouriaghli et al. (2023). This informal drop-in space allows students to receive mental health information without expectation and has been identified as being effective in engaging male students with higher conformity to maladaptive masculine traits, higher self-stigma, and less positive attitudes toward help-seeking. This suggests that informal spaces can improve engagement among groups that may typically avoid formal information sessions.

### Transparency and Collaboration

Seidler, Rice, Ogrodniczuk, et al., (2018) have highlighted the impact of the *transparency* and *collaborative* nature of the approach being taken. In their recent review of the literature, the authors emphasized that a key approach to engaging men is by providing a “collaborative, transparent, and strength-based framework for treatment that promotes empowerment or autonomy over dependence.” This raises questions regarding how much collaboration and transparency there is in mental health interventions for young people.

Effectively adapting these theories to adolescent boys and young men is particularly important, with early intervention seen as a key approach to improving long-term outcomes. Adolescent help-seeking is an important theme in current research on a school-based mental health intervention for 16- to 18-year-olds. Day-long stress workshops, originally developed by Brown et al. (2000), and adapted for adolescents by Sclare et al (2015), are being investigated in U.K. schools between 2021 and 2024. These workshops aim to be accessible to all groups (including male students) and destigmatizing, using non-stigmatizing terms like “stress,” rather than diagnostic terms such as “anxiety” and “depression.” They also use a self-referral system to increase access for “under-served” groups, as reported in previous research (Brown et al., 2004). Despite their effectiveness in reaching under-served groups, such as ethnic minority communities, there is still a gender disparity in workshop participants, with 81.3% being female. Further identification of successful engagement approaches is needed to reach more adolescent boys and young men.

Drawing upon the theories from the adult literature, potential approaches to engage adolescents into the day-long workshops were first discussed with young men from a local Patient and Public Involvement (PPI) group. Four facilitating factors emerged as the most pertinent: positive masculinity and role model; tailoring (and destigmatizing) language; trust building; transparency and collaboration. Therefore, this study aimed to explore these four engagement methods as well as gain views of groups in two regions about these factors in relation to attending the day-long workshops.

Therefore, the aims of this research were:

1. To test if the theories and methods about facilitators and barriers developed for older adult men would be applicable to adolescent young men.
2. To explore the attitudes of adolescent boys toward engagement in day-long workshops that were about to be run in London and Bath.

## Participants

Two focus group meetings were run: one in London and the other in Bath. The participants came from two organizations: male members of a youth panel at a young person’s organization in London, and members of a rugby foundation in Bath. Across these two organizations, 12 male adolescents aged between 16 and 23 were recruited. Purposive sampling was utilized to ensure we recruited male participants within this age range: information sheets were provided to suitable participants via their organization staff and interested individuals were asked to come forward. Ethical approval was obtained from King's College London Research Ethics Committee (MRA-19/20-20581). Written, informed consent to participate was obtained from all participants. It was explained to each participant that any information that indicated they were a significant risk to themselves or others would need to be passed on to the safeguarding staff at their organization.

## Method

Focus groups were chosen as the method to explore the attitudes of adolescent boys toward engagement in the workshop for two reasons. The approach of using an informal and supportive group environment can enhance the discussion of personal issues, resulting in a richer pool of information that may not be captured using individual interviews (Gill et al., 2008; McLafferty, 2004). Furthermore, group discussions not only provide the views of the individual but can also capture group attitudes and norms, which are essential to understand when considering how to engage adolescent boys in a workshop, where information and recruitment sessions (as well as the intervention itself) are often held in group situations.

A semi-structured topic guide was used for each focus group. The four topics were put forward to the participants for discussion in relation to day-long stress workshops that were about to be run.

These were:

1. Can male role models, and other positive masculine figures, be utilized to normalize help-seeking and reduce self-stigma to increase engagement?
2. Can destigmatizing information be framed to emphasize positive masculine traits, such as problem-solving and building skills for life, to increase engagement?
3. Can trust building initiatives encourage help-seeking behavior?
4. Can the feeling of transparency and collaboration increase engagement?

Participants were encouraged to discuss how acceptable and effective these different approaches could be in relation to attending a day-long stress workshop as well as barriers they perceived. As all focus groups were conducted online, a tool called “mentimeter” (www.mentimeter.com) was used, allowing participants to submit answers which then showed up as an anonymous “note.” Once participants had finished submitting their answers, group discussion was facilitated to encourage richer detail to be shared regarding each “note.”

## Procedure

Focus groups were conducted online using Zoom. They were facilitated by the lead researcher (male), a male university student from an existing PPI partnership organization, and a local youth worker (male). The project was led by the lead researcher and the PPI member. The PPI member provided relatability to the participants and helped plan the consultations. During the consultation, the lead researcher and PPI member took detailed notes on what was discussed, as well as the names of participants who had spoken. Note-taking was chosen as the preferred data collection method, as we wanted to encourage maximum trust and openness in these discussions. As discussed by Nordstrom (2015) and Rutakumwa et al (2020), the recording device can have an influence on the interview itself, particularly with groups that may be reluctant to share information due to fear of stigma and shame. Furthermore, at the time of conducting this research the only recording method available via Zoom appeared to be recording the video of the meeting, rather than audio only. Given the potential reluctance of the participants to openly share their views (due to perceived stigma), it was decided that rigorous note-taking was a better option. All recorded notes were stored digitally in password-protected folders on the university storage network.

Each focus group opened by broadly asking the individuals to discuss (a) what issues stopped them taking part in interventions such as day-long stress workshops (“What do you think might put you off, or make you feel unsure, about taking part in a workshop like this?”), and (b) what could be done to help facilitate their participation (“Is there anything you think could be done to overcome these issues and make you want to take part?”). Following this, participants were encouraged to share their views on the specific questions identified regarding engagement.

## Data Analysis

Content analysis (Mayring, 2000) was used to analyze the data. Data were initially collated according to each factor. For each factor, a written record of each point raised was recorded during the session using the mentimeter tool. Further notes were recorded by the researchers present at the consultation during group discussion of each mentimeter answer. These notes were then compared and combined, along with the mentimeter responses and analyzed using content analysis (Mayring, 2000). Concepts were initially defined deductively using the research aims and proposed theoretical approaches. The data were then manually coded into themes. The themes were refined into categories and subcategories. Each subcategory was then analyzed for frequency to give an indication of the prevalence/popularity of these categories and subcategories.

## Findings

The sample consisted of 12 young men who volunteered to take part in the focus groups. The average age of the sample was 18.3 years (*SD* = 1.65, range = 4.91), with 5 (42%) participants from London and 7 (58%) participants from Bath. The themes, categories, and subcategories arising from these consultations are summarized in Table 1.

**Table 1.** Core Themes, Categories, Subcategories, and Number of Quotations From Young Person Consultations

Core theme	Category	Subcategories	*n*
Facilitators			
Transparency and collaboration	Transparency	Why we should take part; What will happen during the workshop; Don’t trick us; Speak on our level	12
	Collaboration	Collaborative approach during workshop	4
Building trust	Confidentiality and non-victimization	Assurance of confidentiality; Agreement among participants that things won’t be shared; Choice of peers to attend with	5
	Increased presence	Ways to find out more over time; Get more familiar; Have information available	4
	Informality	Non-classroom environment; Drop-in space; Make it more relaxed; Make it appealing	5
Positive role models	Expert	Psychologist; Scientist; Project leader	5
	Relatable prior participant	Someone who has experience the workshop; Someone who has benefited from this type of thing; Someone I can relate to who knows about this	4
	Strong figure	Respected individual; Sports person	4
Destigmatizing language	Skills and outcome	Skills I’ll learn; What the outcome will be; How it will help me	8
	Excuse to visit	Refreshments as an excuse to go; Money as an excuse to go; Missing class as an excuse to attend; Going because they need more males	5
Other facilitators	Getting help we need	Help with anxiety; Help me control emotions	3
Barriers to engagement	Prejudices	Self-Stigma / Shame; Social Stigma	12
	Existing symptoms	Increasing existing anxiety; Increasing feelings of shyness	4

### Facilitators

The most common themes (in order of frequency in the discussions) were transparency and collaboration (*n* = 16) and trust building (*n* = 14). Positive male role models were mentioned 13 times, and destigmatizing language was also mentioned 13 times.

#### Transparency and Collaboration (n = 16)

The respondents strongly asserted how important transparency and honesty was to them regarding the reasons for the workshop taking place, what will be involved, and what the outcome might be.

##### Transparency of Participation and Outcomes (*n* = 12)

Being transparent regarding the aims of the workshop and the need for boys to attend was considered very important. Several participants expressed the view that when teachers or other leaders are trying too hard to “convince” or “trick” students into attending something like a workshop, it is picked up very easily by young people and can look desperate/off-putting. One participant noted that “Trying too hard to ‘convince’ or ‘trick’ us into attending is picked up very easily and can look desperate and be off-putting” (Participant 1, Group 1). Another suggested “Just be upfront with us, it’s easy to tell when its fake. Speak to us on a level” (Participant 3, Group 2).

It was suggested that clear and honest presentation of the information, that does not make young men feel patronized, is much more likely to maintain their attention and gain their trust. “Be honest about why you are doing it” (Participant 4, Group 2); “Don’t make it what it’s not” (Participant 2, Group 1).

Participants also expressed that if they had a clear and realistic idea of what it would be like to take part, what they could expect the outcome of taking part to be, and why they were being offered it, they may be more likely to take part. One participant suggested it was important to “tell us what people have gotten out of it” (Participant 1, Group 2). Another noted “it would be good to hear practical examples, e.g., ‘I joined the workshop, and this is what I got from it’” (Participant 5, Group 2).

Finally, it was suggested that often when young men are offered to take part in extra activities such as this at school, they are not given all the information.

Can’t you just be super honest in the information? Can you just say, “we want more boys to take part?” This may be more appealing otherwise it can feel patronising if you are dishonest and seem like they are trying to trick us. (Participant 4, Group 2)

##### Collaboration (*n* = 4)

When asked whether they would value a collaborative approach with the workshop leaders and what this would look like, most participants were focused on what type of individual they would feel comfortable interacting with during the workshop. The most popular suggestions centered around the relatability of the individuals presenting the workshop. “A person I can relate to, who is similar age and has been in this position. Would be better to hear from someone who is actually real and not faking it” (Participant 2, Group 1).

Respondents suggested that they would feel most trusting of a workshop led by an expert with experience but would also feel more inclined to contribute and be involved if they were working in collaboration with an individual closer to their age who they could relate to and who may understand their experiences well.

I would like to see a younger relatable person working alongside a psychology expert, so I could have someone that understands me but also someone who knows everything. Could be co-run by a psychologist as well as somebody else the same age as us. (Participant 6, Group 2)

Another participant noted “I agree, that would be good, someone that understands our situation more and the scientist—someone who you can chat to on the same wavelength, but also someone with more wisdom” (Participant 4, Group 2).

With regard to transparency around the mental health of the workshop leaders, researchers, or teachers at school, the participants were not enthusiastic that hearing this information would particularly help engage them in the workshop. “Not necessarily the expert or whatever but could be good to hear about another student’s first-hand experience” (Participant 4, Group 2).

#### Trust Building (n = 14)

##### Confidentiality and Non-Victimization (*n* = 5)

One point raised consistently was confidentiality and the fear of being victimized by peers due to participation. Participants felt that they (and other students) would be concerned about (a) the information they had shared in any group session being used against them by peers, and (b) their attendance being obvious to other students who weren’t attending. Respondents felt that assurances this would not happen would be an important factor in building trust and convincing them to potentially attend. One participant suggested, “I might go if you could make sure no-one shares what I say” (Participant 5, Group 1). Another noted, “I’d also be put off by leaving lessons and having to explain myself. Might be better if it was mandatory” (Participant 3, Group 1).

It was also felt that they would be more encouraged to attend if they got to choose which students to attend with. “Talk to people about who they would like in their session. Smaller groups with friendly faces would be better” (Participant 4, Group 2). However, some felt it would be impossible to achieve enough confidentiality in a group setting, and therefore boys may struggle to feel trusting of a group workshop. “I think I’d just rather it was one-on-one and more private” (Participant 1, Group 2).

##### Informality (*n* = 5)

When asked what type of environment would encourage them to come and find out more about the workshops, they emphasized some level of informality. It was suggested that being able to get information without pressure to attend could convince them to find out more. Suggestions such as “non-classroom spaces,” and even “places outside of school” were made. “Informal but not too informal would be good—Some people might not like the classroom vibe. Or having a day out to tell you about it and get away from school” (Participant 4, Group 1).

When asked what type of space they would consider attending to find out more about the study, participants re-emphasized the need for an excuse to attend the space, such as a relaxing and comfortable environment, or refreshments. It was also suggested that if a group of friends were there together, they may feel less self-conscious about volunteering to take part because they would feel it was more acceptable to each other. “A place with comfy furniture would be good to relax and talk in” (Participant 4, Group 1); “Games and snacks could be a good reason to go with friends and be less self-conscious about going alone” (Participant 2, Group 2). Another suggested a sort of “pop-up” stand that could be discreetly visited in passing. “A relaxed space at school could be good—somewhere where you have to walk past maybe, pop up stand with stuff to do” (Participant 3, Group 2).

##### Ongoing Increased Presence (*n* = 4)

Some participants suggested that providing availability of information about the workshop in a more continuous manner would potentially normalize it and give the students a greater feeling of trust in taking part. “If it feels more part of everyday school stuff then people won’t care so much who attends” (Participant 1, Group 2). It was suggested that this familiarity and the ability to find out more about the workshop easily, through ongoing access to information via various sources, would make them feel more comfortable in engaging with the workshop.

Have social media maybe or having website or stuff on the school social media channel or posters in school (because most students don’t follow their school online)—it would be better to be able to find stuff out first without having to ask people. (Participant 2, Group 1)

Interestingly, the participants did not express any strong opinions on whether the informal spaces, or any other type of recruitment initiative, were male-only spaces or mixed-gender.

#### Positive Role Models (n = 13)

When discussing which types of individuals could help make the workshop feel more acceptable to attend, in contrast to expectations that a high-profile celebrity would be most persuasive, the most common response was that they would like to hear from an expert in the topic. “I’d rather someone who knows the topic; someone I look up to who can tell me what will happen” (Participant 7, Group 2).

The respondents explained that they would feel more assured by hearing information from someone who is considered an expert and suggested that this would make the topic feel more serious and potentially more acceptable to be interested in, as well as giving them confidence that the information provided was important. “I’d be more interested if I got to hear from a scientist or whoever who knows about this stuff properly” (Participant 1, Group 2). “It would feel more like we’re going to learn something important if it was an expert that was advertising it” (Participant 4, Group 1).

Some respondents suggested that hearing from other individuals that have experienced the workshop could be helpful, with an emphasis on understanding the experience of attending and what effect it had on them. “Hearing from a person I can relate to, who is similar age and has been in this position” (Participant 1, Group 1).

Strong individuals were suggested by some, who explained that a figure associated with strength would reduce the idea that a workshop such as this is for weak students. Some pointed back to their suggestions of the workshop psychologist/scientist as this figure, while others suggested a sport-based individual (potentially a sports teacher) as an example of this. “Sports person—stops ideas of weakness, it’s somebody that you don’t see as weak” (Participant 4, Group 2). However, this was also caveated with suggestions that any role model needed to be believable and genuine. “A teacher could go both ways, depends on the teacher. PE teacher could appeal to some, for the ‘lad’ type. But careful not to manufacture them, they should occur naturally” (Participant 3, Group 1). “Role models occur naturally with time” (Participant 4, Group 2).

#### Destigmatizing Language (n = 13)

##### Focus on Skills Obtained / Skills for Life (*n* = 8)

Throughout our consultations, participants clearly communicated that focusing on what skills they could gain from attending the workshop was likely to be a significant factor in whether they considered attending or not. One respondent noted, “I’d like to know what can I get out of it?” (Participant 2, Group 1). Others suggested they would like to know, “If it can give me skills for the rest of my life” (Participant 1, Group 2), and “stats and quotes for what I will learn” (Participant 4, Group 2).

When questioned whether they would be more attracted to skills that emphasized approaches such as problem-solving and resilience over more obvious “mental health” type approaches, such as discussion of emotions, the majority of respondents emphasized their concern was receiving an *honest* account of exactly what they could expect to gain from the workshop and how it might help them in the future. “Just tell us what people have gotten out of it and how it will help us” (Participant 2, Group 1). However, several participants did suggest that an emphasis on positive skills they could gain from the workshops as would likely be more encouraging to their engagement: “Focusing on aspirations in life etc. may draw in me and other students that would be less up for going to a mental health workshop” (Participant 7, Group 2). Conversely, it was also suggested that while focusing the message on problem-solving may draw in some students that would be less inclined to attend a mental health workshop, they also felt it may alienate those who require this type of support. “A ‘life skills’ approach may only appeal to studious types and alienate those who the workshop was originally meant for” (Participant 5, Group 1).

##### Excuse to attend (n = 5)

When asked what other type of information could engage them in the workshop and encourage attendance, it was suggested that if they were given an excuse to attend, it could allow them to justify their attendance to their peers. One respondent noted,

Refreshments—gives you something to do and a distraction making it less awkward. But may attract people who don’t want or need to be there, but also can attract more people. Could also be another excuse of why you went. (Participant 1, Group 1)

Similarly, it was suggested that if there was a clear message that more boys are needed in the workshops, this could also be used as an excuse of helping out—“If you are honest that more boys are needed it might give boys and excuse to say they are helping” (Participant 3, Group 2).

#### Barriers to Engagement

The most common barriers were self-stigma/shame and social stigma (*n* = 12). When asked what would prevent the participants from attending a workshop, almost all responses centered around fear of judgment from peers and the embarrassment of feeling like they need help.

Toxic masculinity is a big thing and some people don’t want to be seen as weak or lesser than other men because there is a stigma around being a strong figure. This would probably be the main reason I would avoid it and my friends would (Participant 1, Group 2).

Participants reported that they would likely be embarrassed about feeling weak, suggesting self-stigma and shame as a barrier to engagement. One participant noted, “Some of us are scared of being judged for opening up” (Participant 4, Group 1). Others suggested, “I’d be embarrassed about feeling weak” (Participant 6, Group 2), and “Makes you more anxious because people will know you have issues” (Participant 3, Group 1).

## Discussion

### Summary of Findings

The current findings provide some support for existing findings for adult men as well as some novel results for adolescent boys and young men. Some surprising findings occurred regarding facilitators to engagement. The most commonly cited facilitator related to “transparency and collaboration.” The second most common related to building trust. The joint third related to promoting positive masculine role models and using de-stigmatizing language. In terms of barriers, the results support existing research linking maladaptive masculinity ideals to increased feelings of self/social-stigma and shame when seeking help for mental health problems in adult men and suggest this is likely the greatest barrier to engagement of adolescent and young adult men.

### Interpretation of Findings

#### Transparency and Collaboration

This was the most common theme and was very strongly advocated in the focus groups. Participants reported how rarely they feel they are spoken to with transparency about extra-curricular events but strongly felt honesty was incredibly important to them in all areas of engagement. This supports the recommendation that engagement efforts should focus on a fully transparent treatment process (Seidler et al., 2020). Respondents felt more positive about working in collaboration with a younger, more relatable individual as well as an older “expert” who could provide the required knowledge for both engaging into activities, and for the intervention delivery. Participants expressed a preference for working with younger and more relatable individuals who could understand them easily, which could be an effective strategy for engaging adolescent young men, as they may feel more comfortable and understood by someone who is closer in age and can relate to their experiences. Having a younger person as a link between themselves and the intervention leader was seen as potentially beneficial. This suggests that a combination of youth and experience in intervention delivery could be seen as valuable by adolescent young men; older practitioners could provide the necessary expertise and guidance, while younger individuals could help establish rapport and understanding. Research in adult men has also demonstrated that supportive rapport with the practitioner is seen as a facilitator to engagement (Yousaf et al., 2015).

The theme of transparency and collaboration was noted to transcend all the other themes identified in the focus groups. This suggests that transparency and collaboration could be important factors that need to be integrated into various aspects of mental health interventions for young men, such as communication strategies, intervention delivery, and practitioner–patient relationship building.

#### Trust Building

Building trust has been demonstrated as an extremely important factor to engaging men in mental health treatment across ages (Gulliver et al., 2012; Rickwood et al., 2005; Seidler, Rice, Ogrodniczuk, et al., 2018). Fears around confidentiality were a major theme in our focus groups, with many participants reporting that fears of non-confidentiality and subsequent victimization from peers would prevent them from engaging with the workshop. Fears around confidentiality have been identified to present a major obstacle to achieving high-level trust in young men (Lynch et al., 2018). A clear link between trust and confidentiality in young people has been reported (Gonzalez et al., 2005; Jones et al., 2017; Lindsey, & Kalafat, 1998; Wilson & Deane, 2001), with trusting relationships built upon confidentiality.

Another approach suggested increasing trust could be gained by ensuring the individual knows what to expect, through prolonged presence of the workshop at school. They also liked this being informal to potentially reduce fears around victimization. The idea of normalizing such an intervention through low-pressure engagement could prove successful as it has in early studies with university students (Sagar-Ouriaghli et al., 2020). Furthermore, these findings around building trust also tie in with the use of colloquial language; several participants felt it would be very difficult to achieve enough confidentiality in a group setting. Reframing interventions with less stigmatizing and more relatable language, such as referring to a workshop as a “skills for life” workshop instead of a “depression” workshop, may reduce fears of confidentiality breaches and disclosure.

#### Positive Role Models

An interesting finding from these focus groups was that the young men did not suggest that “celebs” would be helpful as role models. Instead, they preferred “experts” whose respected knowledge was difficult to argue with, representing an action-oriented, problem-solving approach which has been reported to engender feelings of strength and empowerment in adult men (Seidler, Rice, Oliffe, et al., 2018). They indicated they were less keen on a perceived “unstructured” approach in which emotions and feelings are readily discussed, which has been reported to put adult men off seeking help (Addis, & Mahalik, 2003). Another role model was a strong and respected figure (such as a sports teacher), who could be effective in encouraging involvement and acceptability, supporting the existing literature around protecting masculine ideals (Seidler et al., 2020). Having a sportsperson, who is seen as fitting the masculine ideal, honestly discuss how they would approve of attendance to this type of workshop could reduce the fear that seeking help reduces one’s ability to fulfill masculine ideals. However, as illustrated in the findings, any role model must be genuine and believable.

Finally, a relatable (younger) role model is also consistent with literature around youth mental health. It has been reported that young people prefer receiving help from those who have lived reality of a young person’s experiences (Cniro et al., 2005; de Anstiss & Ziaian, 2010; Persson et al., 2017). Thus, young men will respond with greater engagement to information from an individual they can associate with, who they feel has been in their position, and may talk confidently about their own help-seeking. It is interesting to note the wording of participants’ responses around this type of role model, often referring to “someone who has done it” or “been in this position.” It could be suggested that young men typically like to see some inspiration; that these problems can be overcome, and that seeking and engaging in help actually works. Peers can have a significant influence on adolescents, and having respected peers who have sought help for mental health issues and can openly discuss their experiences may serve as powerful role models for encouraging help-seeking behaviors. However, the findings also suggest that these young men preferred role models who represent an action-oriented, problem-solving approach. Therefore, it may be important to utilize role models who (to some extent) can portray a proactive and solution-focused approach to mental health, which aligns with traditional masculine ideals of strength, independence, and self-reliance.

#### Destigmatizing Language

Destigmatizing language can be used to highlight practical aspects of the intervention. Consistent with the PLACES model (Brown et al., 2022), which emphasizes the gains from attending rather than the deficiencies, most participants wanted to hear about the positive effects the workshops could have, rather than their deficiencies that need to be treated, which would likely lead to greater engagement. This resonates with the current literature around how men approach mental health services; the uncertainty of expectations and outcomes hinders active participation. This could be viewed as a “cost–benefit analysis” that takes place, whereby the cons of factors such as stigma, loss of masculine ideals, and time are weighed up in relation to the pros, such as benefits, improving well-being, free food, a day off school. Overall, if an intervention has fewer cons and more pros, it would be more likely to have good engagement. In this case, it is likely that emphasizing learning skills to help them succeed could improve engagement due to its ability to “protect” the threat value of stigma about accessing help (Pedersen & Paves, 2014; Topkaya, 2014).

Another possibly less expected finding was the relatively popular suggestion that providing an excuse to attend might help reduce feelings of negative judgment from peers. This again could be perceived as a “pro” to workshop attendance and be seen to reduce the threat value. There is limited specific literature around providing young men with an “excuse” to seek help; however, this finding does support results showing fear of judgment to be a major barrier to help-seeking (Rice et al., 2016). In fact, in a focus group study, Sagar-Ouriaghli et al. (2020) reported that when discussing strategies to “sensitively engage male students” a subtheme of “providing an incentive to attend” emerged, which appeared to act as a mechanism to protect male vulnerability. Therefore, particularly in an open format such as group workshops, providing reasons to attend that may protect the sensitive masculine ideals of adolescent boys and young men could provide a novel method to boosting engagement.

## Future Research

Further research around transparency and collaboration and of building trust in engaging both adult and adolescent young men could be very important. Testing the ideas empirically will also allow them to be more robustly evaluated. Further research exploring the impact of transparency and collaboration in mental health interventions for adolescent young men may involve conducting experimental studies comparing different levels of transparency and collaboration in intervention delivery, communication strategies, and practitioner–patient relationship building, against outcomes such as engagement, satisfaction, and mental health impact. Likewise, developing and testing interventions that specifically target trust-building mechanisms may provide insightful findings. Approaches such as prolonged presence of interventions in school settings, use of informal and low-pressure engagement approaches, and reframing interventions all warrant further examination. Another approach that may be successful in gaining further feedback from this under-served population is the use of anonymous surveys via social media. Research has demonstrated that survey collection using Facebook is effective in reaching those respondents who would otherwise be resistant to sharing their views in approaches like the focus groups conducted in this study (Ellis et al., 2014). As we complete our current clinical trial investigating the effectiveness of the stress workshops in secondary schools, we are using several of these engagement methods and collecting data on take-up among boys.

## Limitations and Considerations

There were several limitations to this research. First, all the young men who participated in our focus groups had volunteered to take part in this study. Second, as these focus groups were centered around hypothetical attendance of a group workshop intervention, the participants were given a description of this group intervention prior to the discussion. Therefore, the responses collected were specific to engagement in this group workshop-based intervention and may be less generalizable to other individual treatments. Finally, although note-taking was conducted thoroughly by two researchers during each consultation, audio recordings of the sessions may have provided a more robust and reliable account of the information shared by the participants.

## Reflexivity

The gender identity of the lead researcher and the youth worker who conducted the sessions as men may have both positive and negative impacts on the research. On the positive side, the fact that the lead researcher and the youth worker are men may have facilitated rapport and trust with the participants. Shared gender identity could have helped establish a sense of commonality, allowing for open and candid discussions about sensitive topics related to masculine identity and mental health. It may have also encouraged young men to feel more comfortable in sharing their perspectives and experiences, leading to richer and more nuanced insights. However, the gender identity of the lead researcher and the youth worker may have also introduced potential biases to the research. For instance, their gender identity could have influenced the framing of questions or the interpretation of responses, potentially shaping the findings in a particular direction. Moreover, the gender identity of the researchers may have influenced the willingness of some participants to disclose sensitive or stigmatized experiences related to mental health, due to concerns about conforming to societal expectations of masculinity. Steps were taken to ensure a reflexive approach during this research process: The lead researcher and the youth worker engaged in regular discussions and critical reflection to minimize these biases and ensure a balanced approach in data collection and interpretation.

## Conclusion

This study sought to identify facilitators and barriers to help-seeking in adolescent boys and young men. When comparing different engagement approaches, transparency and collaboration is the most common theme, followed by building trust. Role models and destigmatizing language were the third most common themes. Unsurprisingly, perceived threat to masculine identity (in the form of self and social stigma) is the main barrier to psychological help-seeking in this population. These results now require testing in experimental studies.
